# Cohort Profile Update: The 45 and Up Study

**DOI:** 10.1093/ije/dyac104

**Published:** 2022-05-23

**Authors:** Kerrin Bleicher, Richard Summerhayes, Sarah Baynes, Michael Swarbrick, Tina Navin Cristina, Hans Luc, Greer Dawson, Alison Cowle, Xenia Dolja-Gore, Martin McNamara

**Affiliations:** The Sax Institute NSW, Australia; The Sax Institute NSW, Australia; The Sax Institute NSW, Australia; The Sax Institute NSW, Australia; The Sax Institute NSW, Australia; The Sax Institute NSW, Australia; The Sax Institute NSW, Australia; The Sax Institute NSW, Australia; The Sax Institute NSW, Australia; School of Medicine and Public Health, The University of Newcastle, NSW, Australia; The Sax Institute NSW, Australia

Key Features

- The 45 and Up Study (‘the Study’) recruited 267 357 participants aged ≥45 years from New South Wales, Australia between 2005 and 2009 as a resource for researchers and policy makers to source evidence to support better health and ageing.
- Participants have received two follow-up surveys with new survey themes responding to emerging research and policy needs such as disability, social connections, loneliness, housing, economic, health status, access to healthcare and impacts of COVID-19.
- Data linkages allow virtually complete follow-up of all participants—currently 212 050 (79.3%) alive and enrolled. The 2018–2020 follow-up surveys had 97 302 responses (47.3% response rate).
- Over 20 data sets including mortality, hospital and pharmaceutical data are routinely linked to the Study to identify sub-cohorts, risk factors, confounders and outcomes. Biospecimens, genomic and environmental data have been collected.
- The Study was designed as a research resource and has been used by >800 researchers, across Australia and internationally. For enquiries, please contact 45andUp.research@saxinstitute.org.au.

## The original cohort

The 45 and Up Study (‘the Study’) was developed to enhance population health. The Study provides researchers with reliable data on a wide range of exposures and outcomes of public health importance for the ageing population. The original 45 and Up Study profile paper was published in 2008 in the early stage of recruitment, describing the recruitment process and characteristics of the first 36 645 (14%) participants.^1^ The present paper profiles the final cohort of 267 357 men and women enrolled from the general population of New South Wales (NSW) and includes Study updates.

Sampling and study design for the 45 and Up Study were described in Banks *et al.*^1^ Briefly, between 2005 and 2009, individuals aged ≥45 years and residing in NSW were randomly sampled from the Services Australia (formerly the Health Insurance Commission) Medicare enrolment database (MEDB) if they had received medical care within the previous 2 years. MEDB includes all citizens and permanent residents. Residents aged ≥80 years and those living in rural and remote areas were oversampled. Eligible people could also volunteer for the Study.

Eligible individuals were mailed an invitation, an information leaflet, the Study questionnaire, a consent form and a reply-paid envelope. Participants who completed the questionnaire and provided signed consent to join the Study and have their survey data linked to a broad range of other data sources were enrolled in the Study. In addition, participants consented to being contacted for 5-yearly follow-up surveys and other research projects.

A total of 1 395 174 invitations were mailed and 267 357 participants enrolled. Participants aged ≥80 years and those living in regional and remote areas were oversampled. The response rate was ≥19.2% (95% CI 19.18–19.22) and included ∼11% of the NSW population aged ≥45 years. The exact response rate may be higher as some people who were selected to participate may not have received any material due to an out-of-date address in the MEDB. The final cohort included 265 821 (99.4%) people who joined the Study by invitation and 1536 (0.6%) who heard about the Study and volunteered independently of the invitation.

## What is the reason for the new data collection?

Since the original cohort paper in 2008, an additional 230 712 participants have been recruited and the baseline survey (2005–2009) and two follow-up surveys (2012–2015 and 2018–2020) have been completed. The surveys included core questions to identify changes in socio-economic factors, health, functional status, health behaviours and lifestyle, and environmental factors (Table 1).

**Table 1:** Themes in three major surveys and two large sub-studies

Demographic and social characteristics and other	Health behaviours	General health-related data
**Baseline collection 2006–2009 (*N* = 267 357)**		
Date of birth	Smoking	Disease and surgical history
Education	Alcohol	Family history of illness
Income	Physical activity	Medication
Marital status	Dietary information	Functional capacity
Country of birth	Sleep habits	Psychological distress
Retirement work		Cancer screening history
Social connectedness		Falls
Ancestry		Oral health
		Skin pigment/response to sun
		Reproductive history
		Incontinence
		Prostate symptoms
**Wave 2: 2012–2015 (*N* = 142 548)**		
Household	Sun protection	Pain
Housing	Sedentary behaviour	Sexual function
Carer responsibilities	End-of-life planning	Allied healthcare, telehealth use
Blood donations	Bowel cancer screening	Irritability
Means of transport	Vaccination—flu, shingles, whooping cough	Healthcare continuity, accessibility, patient experiences
Experience of violence		
**Wave 3: 2018–2020 (*N* = 97 302)**		
New housing questions	E-cigarettes	Blood transfusion
Family history	Quitting smoking	Life satisfaction
Financial stress	Binge drinking	Ability to participate
Use of technology		Aspirin use
		Out-of-pocket health expenses
		Loneliness
		Balance
**Social, Economic and Environmental Factors (SEEF) 2010 (*N* = 60 404)**		
Sexuality		Support required for activities
Religion/beliefs		Fractures
Retirement		Ability to manage healthcare
Social support		Access to healthcare
Major life events		
Financial stress, personal income, dependents		
**COVID Insights: 2020–2022—five surveys (*N* = 32 116 enrolled)**		
Financial security and impact of COVID	Lifestyle behaviour: changes in physical activity, alcohol, sedentary behaviour	COVID exposure, symptoms, infections, testing
Information sources	COVID protective behaviours	**Health service use** including telehealth, allied health, psychological care **Missed/delayed healthcare**: screening, bloods, medications **Impact of missed care**
Health literacy	COVID vaccination—attitudes, perceptions, intentions	**Mental health measures**: De Jong Loneliness scale Kessler psychological distress Duke Social Support Index Uncertainty about the future Concern about COVID infection COVID impact on mental health Resilience index
Major life events	Blood donations	Overall health/quality of life
Work during COVID		Cancer diagnoses and treatment
Carer responsibilities		

New themes have been added to the 5-yearly follow-up surveys in response to evolving policy or researcher information needs. For example, surveys have added questions about end-of-life planning, experience of violence, vaccinations, sedentary behaviours, housing, the COVID-19 pandemic and new factors likely to impact on health (e.g. e-cigarettes). Themes are outlined in Table 1.

New technologies are providing opportunities for cost-effective and more timely collection of survey data. Pre-2015 surveys were conducted by post; however, since then, participants have had a choice to receive and respond to surveys online. The proportion of online responses has gradually increased and in 2020 they accounted for 51% of the core survey responses.

## What will be the new areas of research?

When the 45 and Up Study was established in 2005, the vision was to create a research and policy resource that was adaptable to emerging health issues and changes in the environment. This remains the key aim of the Study and it continues to respond to strategic policy needs, public health information gaps and new research needs. We are seeing a growing interest in testing health interventions, exploring the interaction between phenotypes, lifestyle and genetics, and precision medicine and topical themes like the impacts of COVID-19 and climate change on health.

With the ongoing need for preventive health and management of chronic diseases, the 45 and Up Study is well placed to enable new areas of research. As smoking trends change and new products such as e-cigarettes become available, the 45 and Up Study will enable investigation of cancer risks relevant to today's tobacco-product users and today's tobacco products.

Likewise, geocoded data have begun to be used for novel research on the relationship between green spaces and health, including the impact of neighbourhood green space on sleep,^2^ diabetes,^3^ mental health^4^ and mortality. With changes in the climate, including more extreme heat, more frequent bushfires and droughts across Australia, the Study is poised to support research into environmental impacts on health.

The survey data collected during the COVID-19 pandemic will be linked to other routinely collected sources including hospitalization, mortality, immunization and cancer screening data, for research into the short-term, medium-term and long-term impacts of the pandemic. Public health measures to slow the spread of the pandemic have had differential impacts on the population. The new survey data on missed healthcare, delayed cancer screenings, reduced physical activity, social isolation, financial stress and metal health impacts will support new research that increases understanding and aids planning of health services to focus attention on populations with high healthcare needs and those at risk of poorer outcomes.

Recent advances in the molecular analysis of biological samples have enabled integration with other sources of medical data. As the biospecimen collection is expected to grow in 2022, the continued linkage to other data sources provides the opportunity to identify cohorts of interest and understand the effects of lifestyle factors, health status and genetics on outcomes. This rich resource will facilitate population-level multi-omics research and supports the development of genomic risk tools for early detection for common cancers.

## Who is in the cohort?

### Cohort

The 45 and Up Study has followed the same participants since baseline. As of June 2021, 817 (0.3%) participants had withdrawn and 54 493 (20%) participants had died. The cohort continues to have considerable diversity across age, education and region of residence. Compared with the baseline cohort, the remaining 212 050 participants are older [mean age in June 2021 of 73 (SD 9.1) range 56 to >100 years] and the proportion of females has increased (from 53.6% to 56.5%). There was a greater proportion of participants with education beyond school (up from 43.7% to 47.4%) and Australian-born participants (up from 74.9% to 75.3%) (Table 2).

**Table 2:** Characteristics of 45 and Up Study cohort in June 2021 and at each survey

Characteristics	NSW 2021 population^e^ (aged 55+)	Active participants, 30 June 2021	Baseline 2005–2009	Wave 2 2012–2015	Wave 3 2018–2020
Population	2 417 230	212 050 (79.3%)	267 357	142 548 (53.3%)	97 302 (36.4%)
Deaths		54 493 (20.4%)	Not applicable	11 160 (4.2%)	36 370 (13.7%)
Withdrawals		817 (0.3%)	Not applicable	177 (0.1%)	477 (0.2%)
**Sex**					
Male	1 157 124 (47.9%)	92 216 (43.5%)	124 015 (46.4%)	63 915 (44.8%)	43 559 (44.8%)
Female	1 260 106 (52.1%)	119 834 (56.5%)	143 342 (53.6%)	78 633 (55.2%)	53 743 (55.2%)
**Age (years)**					
Mean age (SD)		72.96 (9.13)	62.76 (11.18)	67.06 (9.74)	70.09 (8.49)
Age range		56–100+	45–100+	49–100+	55–100+
45–54	Not applicable	0 (0.0%)	77 956 (29.2%)	13 659 (9.6%)	0 (0%)
55–64	973 465 (40.3%)	48 575 (22.9%)	85 962 (32.2%)	52 979 (37.2%)	28 975 (29.8%)
65–74	787 479 (32.6%)	83 097 (39.2%)	58 169 (21.8%)	45 535 (31.9%)	40 194 (41.3%)
75–84	461 342 (19.1%)	56 186 (26.5%)	36 986 (13.8%)	22 233 (15.6%)	21 972 (22.6%)
85+	194 944 (8.1%)	24 184 (11.4%)	8276 (3.1%)	8141 (5.7%)	6159 (6.3%)
Missing/invalid**^a^**		8 (0.0%)	8 (0.0%)	1 (0.0%)	2 (0.0%)
**Aboriginal or Torres Strait Islander**					
	33 813 (1.4%)	1560 (0.7%)	1951 (0.7%)	776 (0.5%)	507 (0.5%)
**Country of birth—Australia**		159 652 (75.3%)	200 288 (74.9%)	111 021 (77.9%)	76 147 (78.3%)
**Remoteness** ^b^ ^,^ ^c^ **(ARIA)**					
Major city		109 312 (51.6%)	139 331 (52.1%)	67 649 (47.5%)	48 198 (49.5%)
Inner regional		76 603 (36.1%)	92 872 (34.7%)	46 316 (32.5%)	34 810 (35.8%)
Outer regional, remote and very remote		23 966 (11.3%)	29 895 (11.2%)	12 687 (8.9%)	10 356 (10.6%)
Missing/invalid		2169 (1%)	5259 (2%)	15 896 (11.2%)	3938 (4.1%)
**Index of Relative Socio-economic Disadvantage (IRSD) quintiles** ^c^ ^,^ ^d^					
1 (most disadvantaged)		41 778 (19.7%)	54 659 (20.4%)	25 282 (17.7%)	16 364 (16.8%)
2		44 372 (20.9%)	56 015 (21%)	28 072 (19.7%)	18 909 (19.4%)
3		40 500 (19.1%)	49 641 (18.6%)	25 982 (18.2%)	17 828 (18.3%)
4		37 265 (17.6%)	44 552 (16.7%)	24 620 (17.3%)	17 552 (18%)
5 (least disadvantaged)		44 814 (21.1%)	55 100 (20.6%)	30 491 (21.4%)	21 824 (22.4%)
Missing/invalid		3321 (1.6%)	7390 (2.8%)	8101 (5.7%)	4825 (5%)
**Highest educational qualification**					
No school certificate or other qualification		21 143 (10%)	31 336 (11.7%)	11 544 (8.1%)	6400 (6.6%)
School or intermediate certificate (or equivalent)		45 136 (21.3%)	58 859 (22%)	28 839 (20.2%)	17 530 (18%)
Higher school or leaving certificate (or equivalent)		20 767 (9.8%)	26 104 (9.8%)	13 396 (9.4%)	8830 (9.1%)
Trade/apprenticeship (e.g. hairdresser, chef)		21 873 (10.3%)	29 647 (11.1%)	14 151 (9.9%)	9004 (9.3%)
Certificate/diploma (e.g. childcare, technician)		46 557 (22%)	55 297 (20.7%)	32 562 (22.8%)	23 115 (23.8%)
University degree or higher		53 943 (25.4%)	61 629 (23.1%)	40 645 (28.5%)	31 596 (32.5%)

a Includes eight people with implausible ages (current age >110 years).

b Remoteness areas are aggregates of SA1s that are grouped together based on their average ARIA+ score.^5^

c Based on most recently geocoded address.

d Socio-economic areas are classified according to population-based fifths using the IRSD based on Statistical Area Level 1 of current residence.^6^

e NSW projected population.^7^

Due to the oversampling of older adults and those living in rural areas, the 45 and Up cohort will differ from the NSW population. The proportion of participants identifying as Aboriginal or Torres Strait Islanders was less than the NSW population (0.7% vs 1.4%) for the corresponding age group^7^ (Table 2).

### Survey completion

Thirty-one percent (*n* = 82 728) of the cohort have completed all three surveys, 28% (*n* = 74 394) completed two surveys and 41% (*n* = 110 235) completed only the baseline survey. Regardless of survey completion, data linkages ensure virtually complete and continuous updates of participants’ healthcare, diagnoses and vital status.

Characteristics of participants completing each survey are outlined in Table 3. There was a decrease in the percentage of respondents who spoke a language other than English at home, those living in the more disadvantaged areas and those on a very low income (<$30 000).

**Table 3:** Response rate and additional characteristics of survey respondents

Characteristics	Baseline	Wave 2	Wave 3
Invited	1 395 174	246 306	205 867
Respondents	267 357	142 548	97 302
Response rate	19.2%	57.9%	47.3%
**Smoking status**			
Current smoker	19 019 (7.1%)	5509 (3.9%)	2650 (2.7%)
Former smoker	97 917 (36.6%)	50 380 (35.3%)	33 362 (34.3%)
Never smoker	149 409 (55.9%)	85 238 (59.8%)	60 085 (61.8%)
**Alcoholic drinks per week**			
0 drinks per week	86 657 (32.4%)	44 399 (31.2%)	31 004 (31.9%)
10 or less drinks per week	117 848 (44.1%)	65 729 (46.1%)	45 484 (46.8%)
11 or more drinks per week	55 032 (20.6%)	28 315 (19.9%)	18 345 (18.9%)
**Body mass index (kg/m^2^)**			
**Underweight (<18.5)**	3241 (1.3%)	1927 (1.4%)	1418 (1.5%)
Acceptable (18.5–25)	90 968 (34.0%)	46 274 (32.5%)	31 694 (32.6%)
Overweight (25–29.9)	97 634 (36.5%)	50 622 (35.5%)	35 346 (36.3%)
Obese (30–34.9)	39 385 (14.7%)	21 129 (14.8%)	15 505 (15.9%)
Extremely obese (35+)	15 763 (5.9%)	9141 (6.4%)	6846 (7%)
Missing/invalid	20 186 (7.6%)	13 455 (9.4%)	6493 (6.7%)
**Physical activity—**sufficiently active^a^	170 611 (63.8%)	99 988 (70.1%)	68 253 (70.2%)
**Need assistance due to illness or disability**	**14** **571 (5.5%)**	**10** **819 (7.6%)**	**7865 (8.1%)**
**Chronic conditions^b^**			
Skin cancer (non-melanoma)	68 228 (25.5%)	40 824 (28.6%)	31 300 (32.2%)
Melanoma	14 608 (5.5%)	9994 (7%)	9054 (9.3%)
Prostate cancer^c^	7841 (6.3%)	6359 (10%)	5480 (12.6%)
Breast cancer^d^	7746 (5.4%)	5721 (7.3%)	4893 (9.1%)
Other cancer	16 933 (6.3%)	10 881 (7.6%)	8227 (8.5%)
Heart disease^e^	31 807 (11.9%)	23 799 (16.7%)	18 735 (19.3%)
High blood pressure	95 072 (35.6%)	51 375 (36%)	39 583 (40.7%)
Stroke	8457 (3.2%)	4811 (3.4%)	3179 (3.3%)
Diabetes	23 980 (9%)	14 229 (10%)	10 740 (11%)
Thrombosis	12 354 (4.6%)	6749 (4.7%)	5076 (5.2%)
Asthma^f^	27 197 (10.2%)	17 259 (12.1%)	12 732 (13.1%)
Hayfever^f^	34 567 (12.9%)	22 836 (16%)	17 448 (17.9%)
Parkinson's disease	1682 (0.6%)	1078 (0.8%)	856 (0.9%)
**Multimorbidity** ^g^			
No chronic conditions	115 634 (43.3%)	45 562 (32%)	25 620 (26.3%)
One chronic condition	101 449 (38%)	53 322 (37.4%)	36 142 (37.1%)
Two or more chronic conditions	50 274 (18.8%)	43 664 (30.6%)	35 540 (36.5%)
**Household income per year**			
Less than $30 000	78 243 (29.3%)	33 501 (23.5%)	19 386 (19.9%)
$30 000–$69 999	68 315 (25.6%)	39 959 (28%)	30 007 (30.8%)
$70 000 or more	62 845 (23.5%)	41 758 (29.3%)	29 202 (30%)
Missing/invalid	57 954 (21.7%)	27 330 (19.2%)	18 707 (19.2%)
**Current housing**			
House	203 453 (76.1%)	108 395 (76%)	74 154 (76.2%)
Flat, unit, apartment	28 986 (10.8%)	15 487 (10.9%)	11 086 (11.4%)
House on farm	20 483 (7.7%)	10 381 (7.3%)	6421 (6.6%)
Retirement village, self-care unit	5951 (2.2%)	4016 (2.8%)	3193 (3.3%)
Nursing home	330 (0.1%)	195 (0.1%)	159 (0.2%)
Hostel for aged	912 (0.3%)	280 (0.2%)	97 (0.1%)
Mobile home	2271 (0.9%)	1044 (0.7%)	492 (0.5%)
Other	2501 (0.9%)	1131 (0.8%)	574 (0.6%)
**Other**			
Living alone^h^	–	27 876 (19.6%)	20 674 (21.3%)
Married or living with partner^i^	–	105 180 (73.8%)	70 660 (72.6%)
Language other than English spoken at home	25 495 (9.5%)	9401 (6.6%)	6277 (6.5%)
A carer for sick friend or relative	30 374 (11.4%)	17 401 (12.2%)	11 044 (11.4%)
No time with family or friends in past week	26 881 (10.1%)	11 881 (8.3%)	11 582 (11.9%)
Currently working^j^	144 691 (54.1%)	67 420 (47.3%)	37 501 (38.5%)
Unpaid work	15 687 (5.9%)	8756 (6.1%)	6261 (6.4%)

a Requirement to be sufficiently active is ≥150 min/week of physical activity (where vigorous activity is given twice the weighting of walking and moderate activity) AND ≥5 sessions/week of physical activity.

b Self-reported conditions.

c Males only.

d Females only.

e Baseline question only asked about ‘heart disease’*; Wave 2 and Wave 3 questions asked about ‘heart failure’, ‘atrial fibrillation’ and ‘other heart disease’ and have been combined to get one ‘heart disease’ variable.

f Version 1 of the baseline survey (completed by *N* = 37 172) asked one combined question about ‘asthma or hay fever’. People responding ‘yes’ to this question are excluded here because it cannot be determined which condition they had.

g Conditions included in count of morbidities are self-reported: cancer, heart diseases*, stroke, diabetes, arthritis, asthma and Parkinson's disease.

h Living alone was not asked at baseline.

i Versions 1 and 2 of the baseline survey (completed by *N* = 39 938) permitted multiple response options so baseline numbers are not comparable to other waves.

j Working includes any of: ‘working full-time’, working part-time', ‘partially retired’, ‘self-employed’ and ‘unpaid work’.

Of the invited participants, 97 302 (47%) responded to Wave 3 surveys (Table 3). Seven percent had no school certificate, whereas 32% held a university degree or higher and 6.5% spoke a language other than English in the home. Based on self-reported height and weight, 36% of the cohort were classified as overweight and 23% were obese. Overall, 2.7% of participants reported they were current smokers, 34% past smokers, 66% reported drinking alcohol at least weekly and 26% did not participate in sufficient physical activity by Australian guidelines.^8^

Compared with the baseline surveys, there has been a reduction in smoking in those responding to the 2018–2020 Wave 3 survey (3% vs 7% at baseline). Health problems such as skin cancer, melanomas, diabetes and high blood pressure were more common: 32% of participants reported having skin cancers (26% at baseline) and 41% reported having been diagnosed with high blood pressure (36% at baseline). Consistent with the ageing of the cohort, more participants were seeking help at home (8.1%, an increase of 2.6% from baseline) (Table 3).

## What has been measured?

In consenting to take part in the 45 and Up Study, participants agreed to receive invitations to join focused research projects known as sub-studies to explore issues in more depth. Typically, sub-studies are instigated and led by individual research groups. Eligible participants are invited to join the sub-study of interest and contribute by completing new surveys, donating biospecimens or having other measures taken. After an embargo period, the new data collected through a sub-study become available to other researchers. To date, 19 sub-studies have been conducted involving >100 000 participants. Sub-studies have collected measures of cognition, heart rate, blood pressure, leg strength, assessments of home and neighbourhood, as well as biospecimens and genomic data (Table 4).

**Table 4:** Sub-study data collections

Start year	Sub-study topic	Number of participants
**Biospecimen collections** ^a^		
2009	Link-up—biospecimen collection.^a^ Measured height, weight and waist circumference, BP, HR. Self-reported: height and weight, health, recent health events. Processed biospecimens: plasma, buffy coat, red cells	762
2010	Skin Health Study^a^—biospecimen collection. Self-reported: Height, weight, health, sun sensitivity, lifetime sun exposure, residential history, health history, skin cancer history, health behaviours. Processed biospecimens: plasma, Buffy coat	2571
2018	LinkUp Pathology^a^—biospecimen collection. Self-reported: height and weight, health, recent health events. Processed: serum, plasma, whole blood, DNA, PBMC	1453
**Sub-study data collections**		
2020	COVID Insights—five surveys capturing changes in lifestyle, missed healthcare, health status, wellbeing, attitudes, beliefs and behaviours about COVID-19 vaccinations and COVID-19 prevention activities	32 116
2016	Self-care in Women Living with Chronic Illness—survey	2000
2016	Cancer Recurrence—survey	390
2016	Use of Complementary and Alternative Medicine and Self-care amongst Older Australian Adults with Cardiovascular Conditions—survey	1328
2014	Sexual Wellbeing and Quality of Life after Prostate Cancer—survey	116
2011	Pertussis Vaccine Effectiveness in Older Adults—survey	2487
2010	Diabetes Risk Factors—survey	1210
2010	Social, Economic and Environmental Factors Project—survey	60 404
2009	Successful Transition to Retirement—survey	2579
**Sub-study data currently under embargo** ^c^		
2009	Housing and Independent Living—survey and measures	202
2010	Cardiovascular Risk, E-Couch Depression Outcome—survey	1885
2010	Life Histories and Health—survey	1261
2014	Patients' Experiences Project—survey	7686
2015	Men's Perspectives on Falls and Preventing Falls—survey	25
2016	Maintain Your Brain—detailed cognitive assessment and interventional study to improve cognition^d^	6190
2021	Reconnect—interventional study exploring messaging for those who had stopped statin medication	16 000

^a^ Biospecimens stored at –80 to –196 degrees celsius.

b BP, blood pressure; HR, heart rate; DNA, deoxyribonucleic acid; PBMC, peripheral blood mononuclear cell.

c Please contact Study if interested in these data.

d Data due 2023.

The largest of these sub-studies, the 2010 Social, Environmental and Economic Factors (SEEF Project), was a survey sent to the first 100 000 enrollees in the 45 and Up Study.^9^ It gathered general demographic, health and risk factor data from 60 404 respondents, adding valuable information on: sexual orientation, religious beliefs and faiths, social support, financial hardship, living conditions, neighbourhood safety and access to healthcare. The additional demographic and social information enable research of new sub-cohorts and vulnerable populations.

Other sub-studies have collected biospecimens. To date 4786 participants have contributed blood samples. Aliquots of serum, plasma, whole blood and peripheral blood mononuclear cells have been stored at the NSW Health Statewide Biobank and are available for analysis for approved research projects. To date, 1100 samples from the biospecimen collection have undergone whole-genome sequencing. Of these, 717 were from healthy participants aged ≥70 years and have become part of the Medical Genome Reference Bank.^10^ Several new projects are planned for 2022 utilizing the 45 and Up Study infrastructure to invite participants to donate biospecimens. Projects include a large collection of saliva from 10 000 Study participants as part of the ‘Genomic risk prediction and risk-tailored screening and early detection for common cancers’ project funded by Australia’s Medical Research Future Fund.^11^ Whole-genome sequencing will be performed on the DNA extracted from the saliva samples and these data will be made available for future research projects.

The ability to conduct surveys online has enabled rapid and responsive research during the COVID pandemic. Since July 2020, the 45 and Up Study has collected surveys from 61 700 participants on the impacts of the COVID-19 pandemic. This includes a sub-cohort of 32 116 participants who enrolled in the COVID Insights sub-study to complete a series of five online surveys.^12^ COVID Insights was the largest Australian survey on the impacts of COVID at the time of writing and continues to respond to the rapidly evolving issues associated with the pandemic. It captures self-reported changes in lifestyle, healthcare access, health status and wellbeing, as well as attitudes, beliefs and behaviours about COVID-19 vaccinations and COVID-19 prevention activities. COVID Insights is providing critical information to guide governmental responses to the pandemic. The data is available to researchers and can be linked with other data sources to enable research into the longer-term outcomes of the pandemic.

### Data linkages

At the time of enrolment, all participants of the 45 and Up Study consented to data linkage with other administrative data sources.

Questionnaire data from Study participants are regularly linked with a wide range of administrative data sets, which allows almost complete follow-up of all participants, identification of new diagnoses, healthcare use and outcomes. Commonly, questionnaire data are linked to information on dispensed prescription medication, primary care and specialist health services, mortality, cancer registrations, cancer screening services, hospitalizations and emergency department presentations. Linkages are both retrospective and prospective. Data linkages are regularly performed by the NSW Centre for Health Record Linkage (CHeReL) who have created a Master Linkage Key (MLK) for the Study participants^13^ to enable efficient linkage to NSW data sources.

Confidentialized fine geocoded residential address at baseline and at each follow-up survey has been added, enabling the linkage of valuable environmental information with self-reported and regularly collected health services data.

Ad hoc linkages of questionnaire data and MLK data sets to other data sets can provide insights into more specialized areas—e.g. linkages to infectious disease notifications have informed understanding of herpes zoster, adult vaccination and food-borne illness^14^ and linkage to the National Aged Care Clearing House along with other data sets has improved identification of dementia and its outcomes^15^^,^^16^ and exemplified the benefits of using multiple data sources. A wide range of future linkages are planned, including those going beyond traditional health domains, such as aged care assessment data to provide measures of functional and cognitive limitations that are unavailable in administrative data.

## What has it found?

The 45 and Up Study data have been used in >450 publications that have been cited >10 000 times. The data have been used by >800 researchers across 95 organizations, in >16 countries. Linked with other health data sets, the results from the surveys have been used extensively for epidemiological research into risk factors and incidence of chronic diseases such as cancer,^17^^,^^18^ cardiovascular disease,^19^^,^^20^ mental health^21^ and dementia.^16^ The Study has been used to quantify and characterize social determinants of health and wellbeing,^22^ predictors, changes over time, inequalities of healthcare access and health outcomes^23^^,^^24^ and inform prevention opportunities.

Findings from the Study have informed Australian policies and programmes, including the COVID-19 response,^12^ the NSW Premier’s priorities^25^ and the Greening of Sydney 2030 plan on green space,^26^ development of a NSW Health severe chronic disease management program^27^ and a government’s healthy weight action plan.^28^ In addition, the Study has played a key role in driving tobacco control providing the first large-scale evidence of the impact of smoking in Australia, quantifying the risk of lung cancer associated with light smoking and the effect of reduction in lung cancer risk after smoking cessation compared with reduction, including informing tobacco excise legislation.^29^

## What are the main strengths and weaknesses?

The 45 and Up Study is an independent open-access resource and has had broad-based cross-disciplinary input from national and international academics, policy and decision makers, in what is essentially a co-design process with the research community. This enhances the usefulness of the data collection to inform policy, practice and planning, and supports the goal for all people to have a positive experience of ageing.

The Study is the largest population-based cohort study of its kind in the southern hemisphere and has several unique demographic features. The Study population is relatively heterogeneous, reflecting Australia’s multicultural communities, and has a good spread of responses across most questions. Until recently the Study included the largest older adult cohort of Aboriginal and Torres Strait Islander participants in Australia. The Study’s size and composition allow research into diverse population groups and identification of inequities in healthcare and outcomes.

The Study is also unique among large-scale cohorts internationally in the scope of its ongoing linkages. The data of all participants, including those who do not complete follow-up surveys, can be linked to administrative databases and registers that effectively capture clinical conditions and health services used, cancer registrations and deaths. The combination of large numbers of participants with individual prospective information on exposures, identified through both linked data and surveys, provides a valuable ongoing resource for the investigation of many different causes of morbidity, and mortality and of patterns of health-related service use.

Further strengths of the Study include the oversampling of people aged ≥80 years (with no upper age limit) and those from rural areas, enabling a particular focus on these often under-represented groups. In addition, researchers can safely and remotely access linked health data through a secure computing environment [Secured Unified Research Environment (SURE)]. Other strengths include: the accessibility of the data through the Study’s governance structure, engagement with participants and partnerships with policy agencies and non-government clinical disease organizations.

The growing uptake of communication technologies (e-mail, computers and mobile devices) among participants and support for 45 and Up sub-studies have facilitated a more timely and cost-effective approach to new research. This has enabled efficient rapid response to critical health issues, e.g. during the COVID pandemic.

One limitation of the Study is the lack of large-scale clinical data on measures such as blood pressure, spirometry, anthropometry and cognition, and the limited collection of biospecimens. However, adding further biological samples to the Study over time will enhance its value as a resource. Additionally, Study questionnaires have only been written in English, thereby limiting the initial enrolment and participation to people with sufficient English literacy. Lastly, although participants were derived from the general population, the oversampling of people from regional areas and those aged ≥80 years is both a benefit and a limitation; the Study was not designed to be representative of the general population and therefore does not necessarily provide population-level measures of incidence and prevalence. Regardless, the 45 and Up Study is likely to be one of the most inclusive large-scale cohort studies conducted to date and its findings from groups within this cohort should be applicable to broader populations.

## Can I get hold of the data?

The Study is accessible to researchers for high-quality research that is in the public interest. All research projects require Ethics Approval and data analyses must be conducted in the Sax Institute’s online workspace (Secure Unified Research Environment, SURE). Details of the data access policy and procedures are available at the Sax Institute’s 45 and Up website (https://www.saxinstitute.org.au/our-work/45-up-study/for-researchers/). Researchers can also apply to use the 45 and Up Study as a framework for more detailed data collection (sub-studies) and for intervention studies. Expression of interest forms are online,^30^ together with Study questionnaires,^31^ technical information^32^ and 45 and Up Study publications.^14^ For queries, please e-mail Kerrin Bleicher at 45andUp.research@saxinstitute.org.au.

## Ethics approval

Ethical approval for the Study was provided the University of NSW Health Research Ethics Committee (HC210602) on 25 July 2005 with regular extensions granted, most recently from 9 September 2021 to 8 September 2026. All participants provided written informed consent, returned with the baseline questionnaire before being enrolled in the cohort study.

## Data Availability

See ‘Can I get hold of the data?’ above.
